# Full cervical cord tractography: A new method for clinical use

**DOI:** 10.3389/fnana.2022.993464

**Published:** 2022-09-27

**Authors:** Corentin Dauleac, Carole Frindel, Isabelle Pélissou-Guyotat, Célia Nicolas, Fang-Cheng Yeh, Juan Fernandez-Miranda, François Cotton, Timothée Jacquesson

**Affiliations:** ^1^Service de Neurochirurgie, Hôpital neurologique et neurochirurgical Pierre Wertheimer, Hospices Civils de Lyon, Lyon, France; ^2^Laboratoire CREATIS, CNRS UMR5220, Inserm U1206, INSA-Lyon, Villeurbanne, France; ^3^Université de Lyon I, Lyon, France; ^4^Hospices Civils de Lyon, Centre Hospitalier de Lyon Sud, Service de Radiologie, Lyon, France; ^5^Department of Neurological Surgery, University of Pittsburgh Medical Center, Pittsburgh, PA, United States; ^6^Department of Neurosurgery, Stanford University Medical Center, Stanford, CA, United States

**Keywords:** spinal cord, tractography, fiber orientation distribution, fiber tracking, diffusion tensor (DT) MRI

## Abstract

Despite recent improvements in diffusion-weighted imaging, spinal cord tractography is not used in routine clinical practice because of difficulties in reconstructing tractograms, with a pertinent tri-dimensional-rendering, in a long post-processing time. We propose a new full tractography approach to the cervical spinal cord without extensive manual filtering or multiple regions of interest seeding that could help neurosurgeons manage various spinal cord disorders. Four healthy volunteers and two patients with either cervical intramedullary tumors or spinal cord injuries were included. Diffusion-weighted images of the cervical spinal cord were acquired using a Philips 3 Tesla machine, 32 diffusion directions, 1,000 s/mm^2^ *b*-value, 2 × 2 × 2 mm voxel size, reduced field-of-view (ZOOM), with two opposing phase-encoding directions. Distortion corrections were then achieved using the FSL software package, and tracking of the full cervical spinal cord was performed using the DSI Studio software (quantitative anisotropy-based deterministic algorithm). A unique region of avoidance was used to exclude everything that is not of the nervous system. Fiber tracking parameters used adaptative fractional anisotropy from 0.015 to 0.045, fiber length from 10 to 1,000 mm, and angular threshold of 90°. In all participants, a full cervical cord tractography was performed from the medulla to the C7 spine level. On a ventral view, the junction between the medulla and spinal cord was identified with its pyramidal bulging, and by an invagination corresponding to the median ventral sulcus. On a dorsal view, the fourth ventricle—superior, middle, and inferior cerebellar peduncles—was seen, as well as its floor and the obex; and gracile and cuneate tracts were recognized on each side of the dorsal median sulcus. In the case of the intramedullary tumor or spinal cord injury, the spinal tracts were seen to be displaced, and this helped to adjust the neurosurgical strategy. This new full tractography approach simplifies the tractography pipeline and provides a reliable 3D-rendering of the spinal cord that could help to adjust the neurosurgical strategy.

## Introduction

Advances in magnetic resonance (MR) imaging and the introduction of diffusion tensor imaging (DTI) associated with tractography have allowed the description of the white matter fibers of the brain *in vivo* (Chao et al., 2007; Catani and Thiebaut de Schotten, 2008; Fernández-Miranda et al., 2008; Maier-Hein et al., 2017; Jacquesson et al., 2018). Tractography uses the preferential diffusion orientation of water molecules constrained by tissues to reconstruct their architecture using a mathematical algorithm (O'Donnell and Westin, 2011). However, tractography of the spinal cord remains challenging due to its specific anatomy: a long, thin tube composed of billions of fibers condensed in a very small volume, and surrounded by many structures (cerebrospinal fluid [CSF], vertebral disc, thoracic and abdominal content, etc.) that induce artifacts on diffusion-weighted images (Jezzard et al., 1998; Ruthotto et al., 2012; David et al., 2017).

Developments in both imaging acquisition and post-processing have improved the accuracy and reliability of tractography (Andersson and Sotiropoulos, 2016; Cohen-Adad et al., 2021a; Dauleac et al., 2021), and have highlighted the potential key role of tractography, compared to classical anatomical MR sequences, for a wide range of spinal cord diseases [intramedullary tumors (Choudhri et al., 2014; Egger et al., 2016), vascular malformations (Dauleac et al., 2019a), spinal cord injury (Chang et al., 2010; Rao et al., 2013), syringomyela (Dauleac et al., 2019b), multiple sclerosis (Cruz et al., 2009; van Hecke et al., 2009), cervical myelopathy (Lee et al., 2011)]. Spinal cord tractography is not currently possible in routine clinical practice because of the difficulties in producing tractograms in an accessible way, but more importantly, tridimensional renderings are not yet consistent with the anatomical truth (Dauleac et al., 2020, 2021). Also, tractography results are strongly dependent upon the user who defines the parameters in all the steps of the imaging pipeline, from imaging acquisition to post-processing; this is of particular importance for the design of regions of interest (ROIs) and the elimination of spurious fibers (Roundy et al., 2012). The classical ROI-based method for tractography has many limitations: the manual delineation of multiple ROIs is a time-consuming and fastidious process with a slow learning curve, the ROI placement requires prior anatomical knowledge, ROIs have to be designed on a dedicated software, and the diffusion images on which the ROI are drawn can be affected by artifacts and distortions (De Leener et al., 2017). Moreover, mathematical algorithms can fail to reconstruct spinal cord fibers because of their specific anatomy (lordosis, emergence of spinal roots, and small volume). Therefore, a simple pipeline that would allow an accurate high-resolution spinal cord tractography for imaging and surgical reproducible routine use would be of great interest.

On the basis of our previous experience in brain and cranial nerve tractography (Jacquesson et al., 2018, 2019), we propose a novel practical full spinal cord tractography approach, including successively advised MR acquisition settings, distortion corrections, a unique region of avoidance, generalized q-sampling reconstruction (Yeh et al., 2010), quantitative anisotropy-based deterministic fiber tracking (Yeh et al., 2013), and an attractive tridimensional rendering of the whole spinal cord volume. We aim to introduce this method to the public and broaden its use in routine clinical practice.

## Materials, equipment, and methods

### Participants

**Figure 1:**
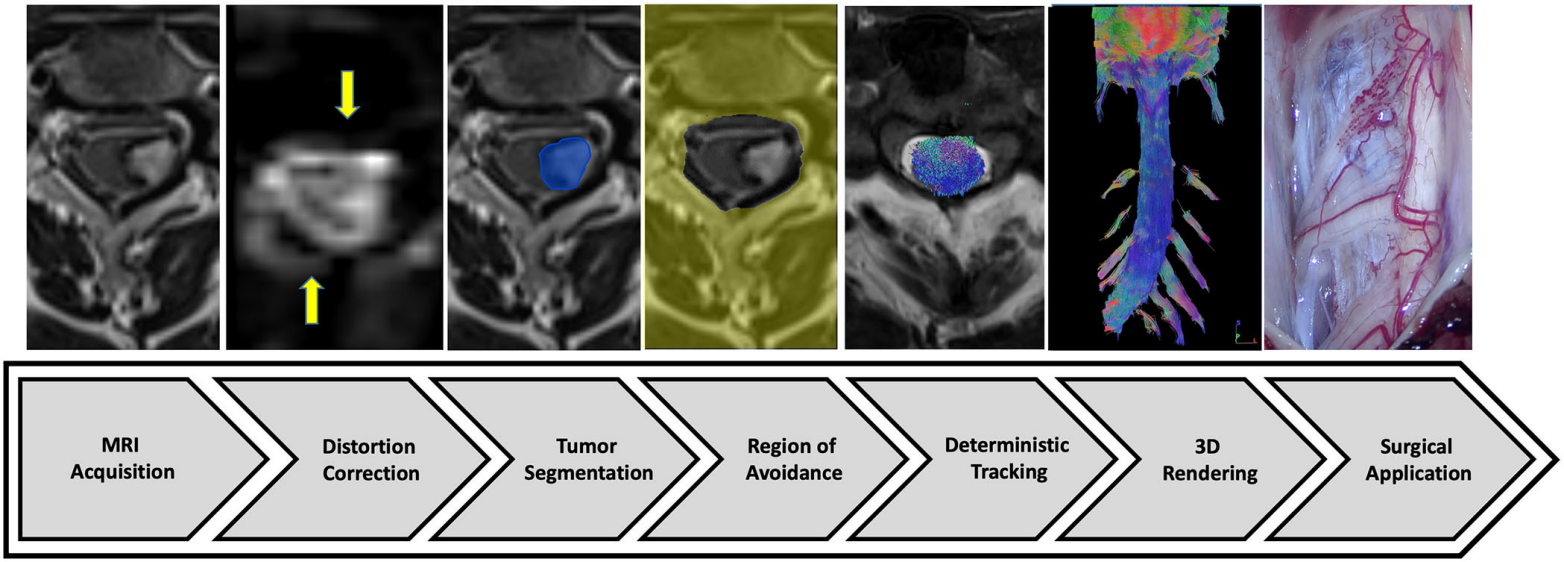
Study diagram. From Philips MRI machine, diffusion tensor imaging associated with T2-weighted imaging (and enhanced T1-weighted imaging if necessary) were acquired (step 1). The post-processing protocol included distortion correction (step 2), tumor segmentation (step 3), and region of avoidance design (step 4). The tracking process (step 5) was performed using the DSI-Studio software to visualize a 3D full cervical spinal cord tractography (step 6) to help neurosurgical management (step 7).

Four healthy volunteers were included in the present study that was conducted at the university hospital of Lyon, France between 2020 and 2021. Three patients were also included: a 51-year-old woman with a left C3–C4 intramedullary cavernous malformation, a 32-year-old man with C3–C4 spinal cord injury, and a 42-year-old man with intramedullary tumor. The institutional review board approved the study and written informed consent was obtained from all participants. The tractography pipeline proposed is composed of seven steps (Figure 1).

### Image acquisition

Diffusion-weighted images were acquired using a 3 Tesla Ingenia Elition MRI machine (Philips Medical Systems, Netherlands) with the following parameters: 32 directions (two acquisitions in opposed phase-encoding directions), *b*-value: 0, 1,000 s/mm^2^, TE/TR: 64/3,220 ms, voxel size: 2 × 2 × 2 mm^3^, no slice gap, coronal plane (10 slides), with fat suppression (SPIR), and reduced field-of-view (180 × 56 × 40 mm): adaptation of zonally magnified oblique multi-slice (ZOOM) sequence. This method exploits the concept of inner volume imaging, and enables the selective excitation and refocusing of a narrow field-of-view while avoiding signal originating from outside the desired field-of-view (Samson et al., 2016). The spinal cord DTI sequence was first acquired with the right → left phase-encoding direction in 3 min 45 s, and second with the left → right phase-encoding direction, without in-plane acceleration (Table 1). Sagittal T2-weighted imaging was added as a reference, or a contrast-enhanced T1-weighted sequence was used in the case of spinal cord tumors.

**Table 1:** Diffusion acquisition and tracking parameters.

	**Parameter**	**Value**
***MRI Acquisition***	MRI Machine	Philips
	Magnetic field	3 T
	Diffusion directions	32
	b value	1,000 s/mm^2^
	Slice thickness	2 mm
	Voxel size	2 × 2 × 2 mm *isotropic*
	Diffusion slice gap	0
	Field of view	Tailored to spinal cord – ZOOM DTI
	Acquisition plane	Coronal
	Phase encoding direction	Right → left and left → right
	TE / TR	Lower as possible
***Tracking***	Distortion correction	Eddy FSL tool
	Software package	DSI Studio
	FA threshold	“default” 0.015–0.045
	Curvature threshold	90°
	Min–max length	10 - 1000 mm
	Step size	0.1 mm
	Reconstruction algorithm	Deterministic
	ROA design	One ROA including the whole spinal cord.
	Filtering	Manual elimination of spurious fibers and false continuation (<15min)

FA, fractional anisotropy; ROA, region of avoidance.

### Distortion corrections

Correction for susceptibility artifacts was performed using the “top-up” tool from FSL (Smith et al., 2004) (Functional magnetic resonance imaging of the brain [FMRIB] Software Library) to reduce inhomogeneity distortions (Andersson and Sotiropoulos, 2016). Motion artifacts and artifacts generated by eddy currents produced during diffusion-encoding gradient application were corrected using the FSL “eddy”. The methodology used herein has been demonstrated to be an essential step to obtain a good spinal cord tractography rendering (Dauleac et al., 2021).

### Fiber tracking

**Figure 2:**
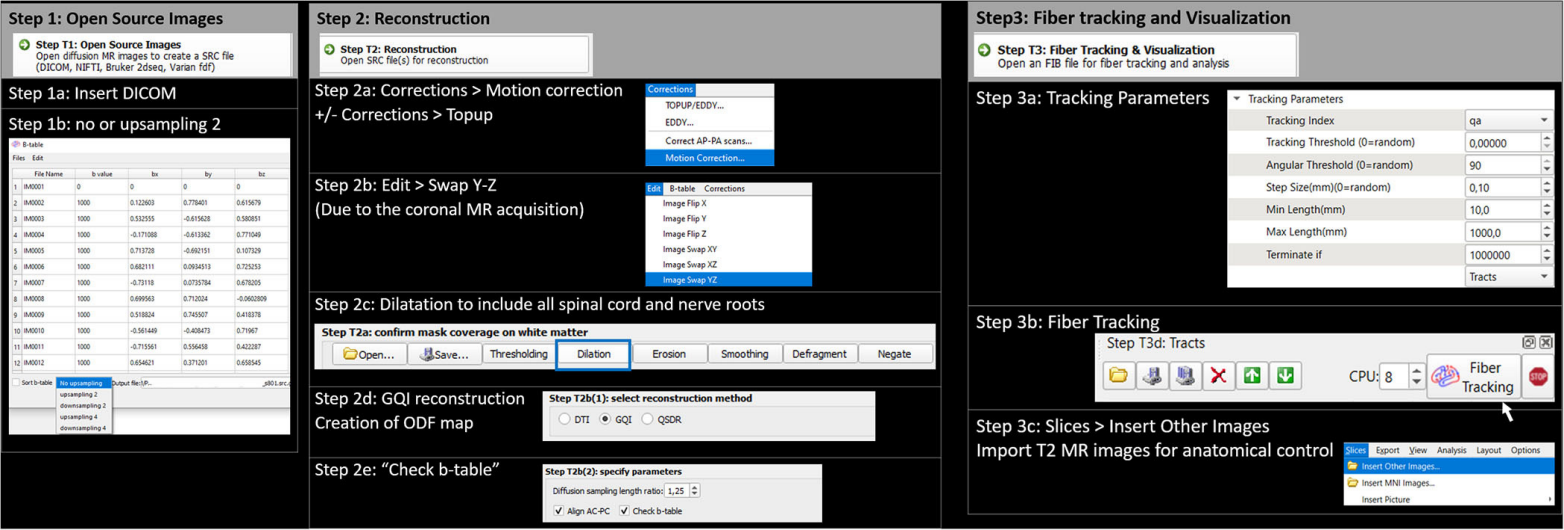
Fiber tracking process using DSI Studio software package: step-by-step. Step 1: Open source images. Step 1a: Insert DICOM. Step 1b: use no upsampling (or upsampling 2). Step 2: Reconstruction. Step 2a: Artifacts correction (motion correction +/– topup). Step 2b: Swap images according to the *Y*–*Z* axis because of coronal MR acquisition. Step 2c: Dilatation of the working mask to include all the spinal cord and nerve roots. Step 2d: GQI (generalized Q-sampling imaging) reconstruction. Step 2e: check the b-table (necessary because of the image swap performed above). Step 3: Fiber tracking and visualization. Step 3a: Use tracking parameters defined in Table 1. Step 3b: Perform fiber tracking. Step 3c: Superimpose T2-weighted images.

A unique ROI volume around the whole spinal cord and enlarged (about 10 mm) to include rootlets in the vertebral canal (Jacquesson et al., 2019) was drawn on the orientation of distribution function (ODF) map before being negated to create a unique region of avoidance. This leads to limiting exclusively fiber tracking to the spinal cord area. Tumor or trauma segmentation was performed manually—slice by slice—on anatomic images. Full tractography was performed out of the region of avoidance that reduced the manual step of multiple ROI design. Quantitative anisotropy was obtained from a generalized q-sampling reconstruction (Yeh et al., 2010). Tractography was performed using the DSI Studio software (Yeh et al., 2013) and a deterministic fiber-tracking algorithm based on quantitative anisotropy to improve accuracy (Yeh et al., 2013). The tractography algorithm used the following parameters: step size = 0.1 mm, fiber length = 10 (min) to 1,000 (max) mm, angular threshold = 90° (to not miss any fibers exiting orthogonally from the spinal cord), and adaptative fractional anisotropy threshold that varied from 0.015 to 0.045 (Dauleac et al., 2020). This fractional anisotropy threshold was selected using a compromise value between maximum anatomical details and minimum “noise”. A total of 50,000 tracts were calculated. Fibers were displayed as 0.15-mm-diameter tubes (Figure 2). Manual filtering of spurious fibers and false continuations was then performed for a maximum of 15 min (Table 1).

## Results

### Cervical spinal cord

**Figure 3:**
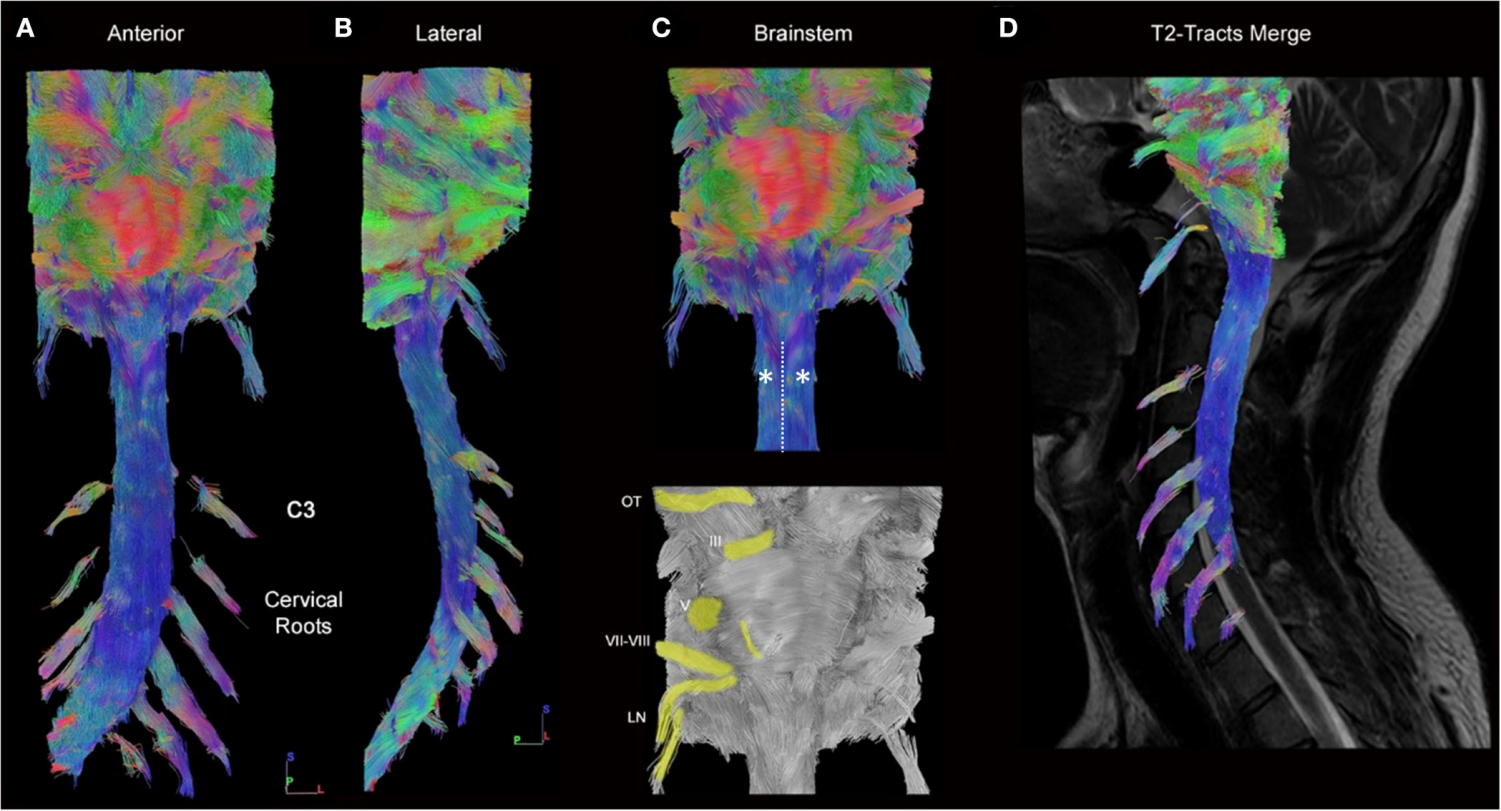
Full cervical cord tractography (healthy subject). **(A)** Ventral and **(B)** lateral views of the cervical spinal cord and brainstem tractography showing the ascending/descending pathways with cervical rootlets. The emergence of the rootlets from the spinal cord can be seen laterally (turquoise). Spinal cord fibers (blue) are well reconstructed on the whole length of the spinal cord in a subject who had an important cervical lordosis. **(C)** From this enlargement of panel A on the brainstem, pontocerebellar fibers are seen (red), cranial nerves (from optic tracts [OT] to lower nerves [LN]), including acoustic-facial bundle [VII–VIII] are highlighted (yellow). The junction between the medulla and spinal cord was identified by its pyramidal bulging (*), and by an invagination that corresponds to the median ventral sulcus (dotted line). **(D)** The spinal cord tractography is superimposed on the sagittal T2-weighted images.

Full tractography depicting the cervical spinal cord was obtained in all participants. The tridimensional view allowed rotation and magnification on demand and displayed the details of the spinal cord anatomy. Because of the directional color code, the fibers of the spinal cord that are ascending and descending appeared in blue (Figure 3). Some roots were seen (Figures 3A,B,D). The cervical lordosis was visualized, and the tractogram mostly ended at the C7 spine level (Figure 3B). At the top of the acquisition box, the brainstem (mesencephalon, pons, and medulla) fibers were tracked. On a ventral view, the junction between the medulla and spinal cord was identified with its pyramidal bulging, and by an invagination corresponding to the median ventral sulcus (Figure 3A). The corticospinal tract midline crossing was recognized just inferiorly. Most cranial nerves, from optic to lower nerves, were also visible (Figure 3C). On a dorsal view, the fourth ventricle—superior, middle, and inferior cerebellar peduncles—was seen, as well as its floor and the obex (Supplementary Video 1). The gracile and cuneate tracts were seen on each side of the dorsal median sulcus. The same aspect was found for all healthy subjects.

### Illustrative case 1

**Figure 4:**
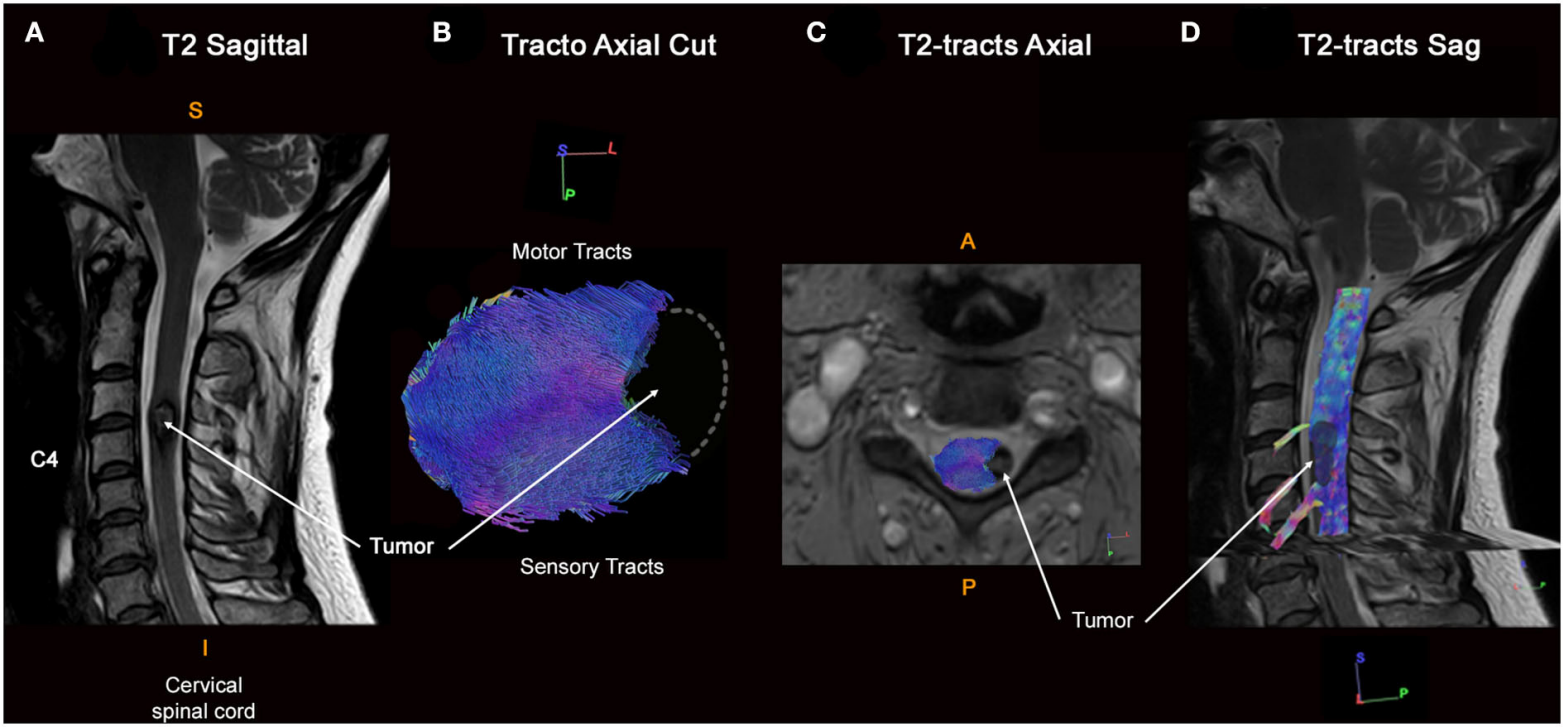
Full cervical cord tractography in a patient with intramedullary cavernous malformation. **(A)** Sagittal T2-weighed images showing the cavernous malformation at the C3–C4 spine level. Cavernous malformation presented an exophytic portion on the left side of the spinal cord, with a hemosiderin crown in a hyposignal T2-weighted sequence. **(B)** Axial view of spinal cord tractography at the C3–C4 level shows that spinal fibers were pushed back and compressed by the cavernous malformation, without fibers around the cavernous malformation (at its dorso-lateral part). Left lateral view of cervical spinal cord tractography showing a notch within the spinal cord repressing spinal tracts. **(C)** Overlay of the spinal cord tractography on the axial T2-weighted MR images. **(D)** Overlay of the spinal cord tractography on the sagittal T2-weighted MR images, with the 3D rendering of the cavernous malformation (in black).

This 51-year-old woman presented paresthesias associated with motor weakness of the left upper limb. The MR images revealed a left C3–C4 intramedullary cavernous malformation, with a hemosiderin crown. However, on axial T2-weighted images, there is uncertainty about the passage of fibers in the latter region. Full tractography demonstrated that there is no fiber within the malformation, so fiber tracts were pushed medially at the exact edge of the hemosiderin crown (Figure 4). This information, in addition to intraoperative neurophysiological monitoring and perioperative findings, led to displacing the myelotomy laterally thus limiting damage to posterior white tracts.

### Illustrative case 2

**Figure 5:**
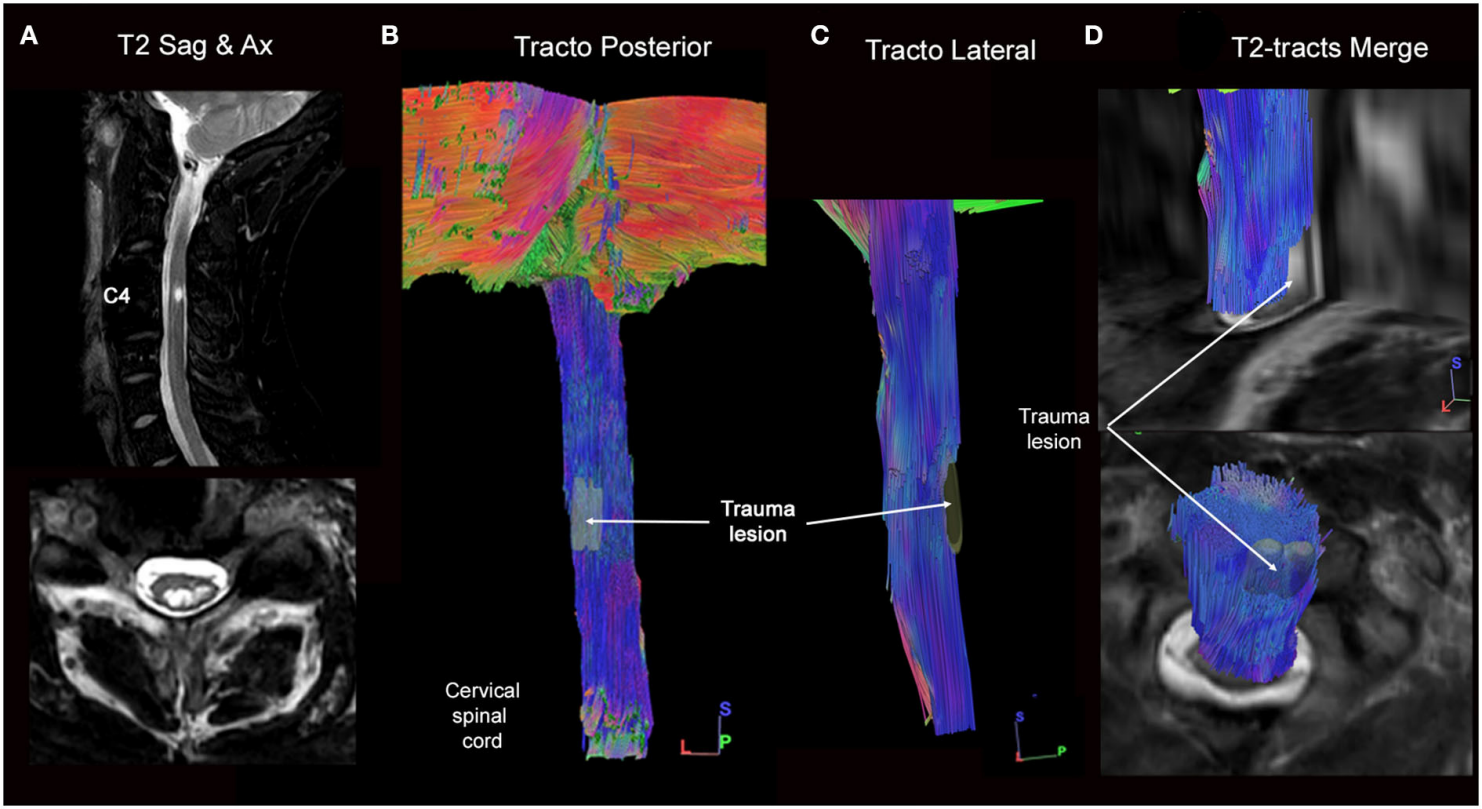
Full cervical cord tractography in a patient with spinal cord injury. **(A)** Sagittal and axial T2-weighed images showing intramedullary hypersignal at the C3–C4 spine level. On an axial image, intramedullary T2-hypersignal is seen on the left dorso lateral part of the spinal cord. **(B)** Dorsal and **(C)** lateral views of spinal cord tractography showing the complete interruption of left dorso-lateral spinal cord fibers. **(D)** Overlay of the spinal cord tractography on the sagittal T2-weighted MR images, where the correspondence between T2 hypersignal and fibers loss was perfectly defined.

This 32-year-old man had a “whiplash-like” cervical trauma followed by a left Brown-Séquard syndrome. Cervical CT and MRI scans revealed a C3–C4 spinal cord injury, disc herniation, and vertebral ligament damage. An emergency C3–C4 anterior cervical discectomy with decompression and fusion was performed. Six months postoperatively, after intensive rehabilitation, the patient was able to walk but presented important proprioceptive ataxia associated with the motor weakness of the left upper limb. Full tractography found fiber interruption of posterior columns of the spinal cord mainly to the left side (Figure 5). This information was used to focus on the rehabilitation program of the patient.

### Illustrative case 3

**Figure 6:**
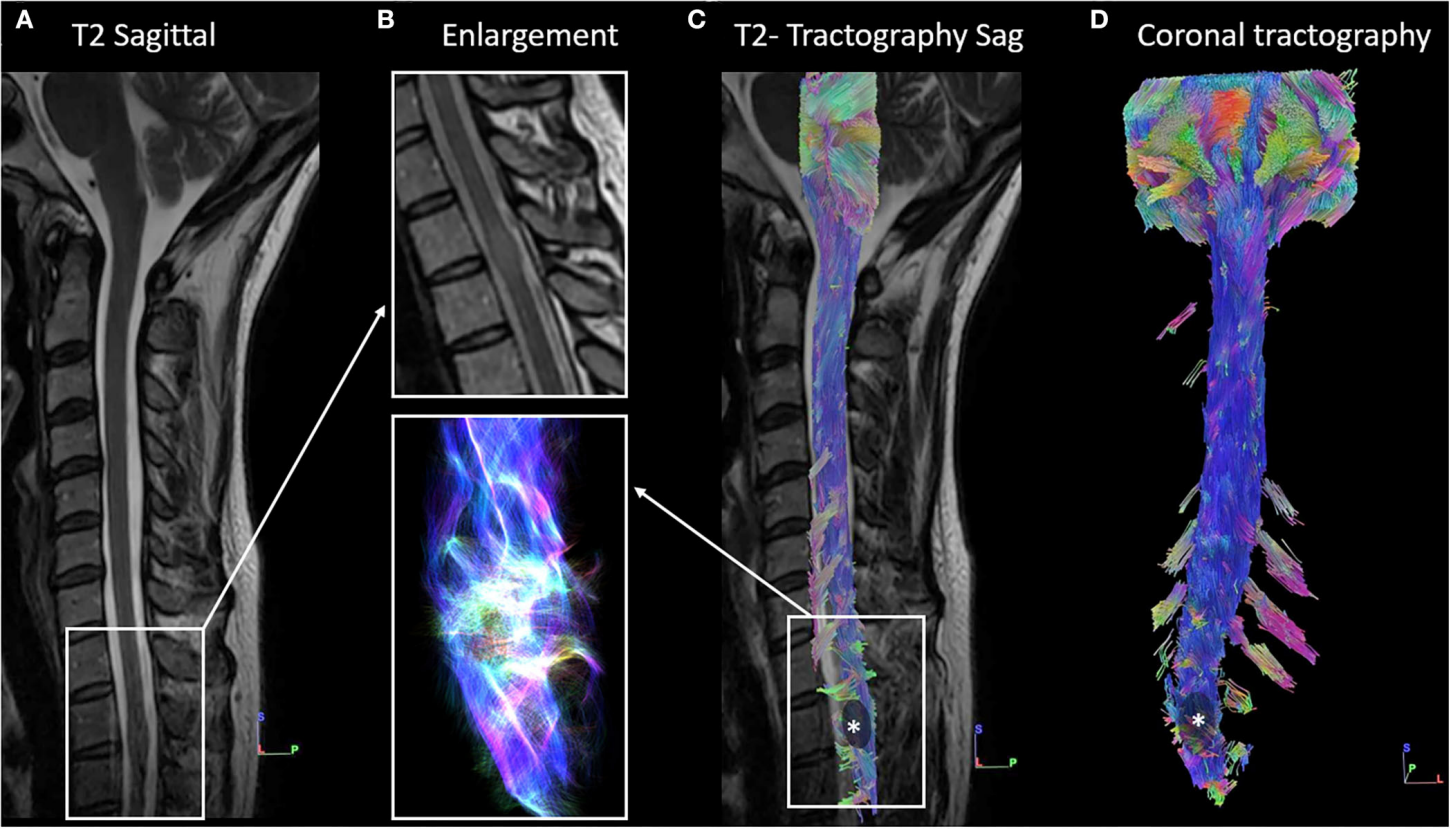
Full cervical cord tractography in a patient with intramedullary tumor. **(A)** Sagittal T2-weighed image showing intramedullary isosignal at the Th2-Th3 spine level, associated with an increase in the volume of the spinal cord. **(B)** 1-Enlargement of the **(A)** at the level of intramedullary lesion, on the T2-weighed image. 2- Enlargement of the **(C)** on fiber tracking, according to a “line” style rendering performed in DSI Studio, at the level of the lesion. It shows warped fibers within the tumor, without safe access for spinal cord surgery. **(C)** Overlay of the spinal cord tractography on the sagittal T2-weighted image confirmed fiber deformation at the Th2-Th3 level. **(D)** Ventral view of spinal cord tractography showing warped fibers at the level of the tumor (in black with *).

This 42-year-old man presented paresthesia with neuropathic pain in the lower limbs. Spinal cord MRI demonstrated an intramedullary lesion in Th2–Th3, without cyst or gadolinium enhancement. The patient underwent extensive investigations that confirmed the absence of any inflammatory, infectious, or neurodegenerative disorders. Full spinal cord tractography showed warped fibers within the tumor, without fiber compression (Figure 6). This added arguments in favor of the diagnosis of astrocytoma. In addition to a low clinical impact, full tractography helped to orient the strategy to surveillance.

## Discussion

This study presents an original full cervical cord tractography approach without extensive manual filtering or multiple ROI seeding. Furthermore, we propose a complete pipeline from MR acquisition to tracking settings that has shown interest in illustrative cases and could be hence used in routine neurosurgical practice.

### ROI-based vs. full tractography

Only a few studies have focused on spinal cord tractography with clinical applications (Ducreux et al., 2006; Ozanne et al., 2007; Granata et al., 2017; Dauleac et al., 2019a), probably due to its complex and time-consuming pipeline, including data acquisition, post-processing, and filtering. In recent studies, investigating spinal cord tractography, either an ROI was placed on the whole spinal cord on an axial section (“single ROI design”) (Ducreux et al., 2006; Ozanne et al., 2007; Granata et al., 2017; Dauleac et al., 2019a), or multiple ROIs were placed on a spinal cord axial plane to exclude gray matter (“multiple ROI design”), and both single and multiple ROI designs have disadvantages (user-dependent and time-consuming). When using ROIs, some authors described the impact of the sagittal balance (i.e., cervical lordosis and thoracic kyphosis) on the spinal cord tractography rendering (Barry et al., 2018; Dauleac et al., 2021), and reported that a greater number of artifacts is observed at the C7 level (i.e., where the cervical lordosis is the most significant) (Barry et al., 2018). Moreover, a critical angle (22°), above which, fibers were not tracked along the entire length of the spinal cord was defined (Dauleac et al., 2021). With regards to the placement of ROI(s), difficulties for tractography of the lower spinal cord, such as the conus medullaris, were reported because of the high condensation of spinal tract ends as well as the emergence of many rootlets (van Hecke et al., 2009; Filippi et al., 2010; Antherieu et al., 2018). Some authors proposed to draw smaller ROIs (Facon et al., 2005; Ducreux et al., 2006; Xiangshui et al., 2011), but this would lead to a loss of fibers tracked because of the minimal resolution of the MR images obtained in routine practice. In addition, ROI placement should avoid areas where partial volume effects, magnetic susceptibility effects, and motion artifacts are important (Facon et al., 2005; Ducreux et al., 2006; Xiangshui et al., 2011; Wang et al., 2012b). To overcome all these issues related to ROIs, we chose to use a unique region of avoidance that includes the whole spinal cord; furthermore, this eliminates both inter- and intra-individual variability of ROI design (Van Hecke et al., 2008).

### Toward routine spinal cord tractography

Spinal cord tractography is currently limited to research applications (Chang et al., 2010; Mohammadi et al., 2013; Andersson and Sotiropoulos, 2016; Calabrese et al., 2018; Dauleac et al., 2021). The obstacles are multiple: acquisition parameters must be optimized to extract the diffusion signal on a highly selected volume (Dauleac et al., 2020; Cohen-Adad et al., 2021b); a correction of distortions is required to correct susceptibility artifacts because of the presence of various tissue types (e.g., air in the trachea, vertebrae bone, intervertebral discs, CSF, thoracic and abdominal contents) that distort the field around the spinal cord (Barry et al., 2018); scan time has to be acceptable for clinical use. For these reasons, we used a diffusion-weighted sequence in a coronal plane that allows an acquisition focused on the spinal cord in only 10 slides that limited scan time and diffusion artifacts due to thoracic structures. In addition, the field-of-view was reduced by applying a dedicated acquisition (ZOOM- Philips or ZOOMit- Siemens, or FOCUS- General Electrics) to exclude irrelevant components: vertebras, intervertebral discs, larynx, lungs, etc. Another obstacle is that post-processing of images requires specialized training, and to render this accessible to any user we also propose to reduce the user-related steps of deterministic tractography (ROI design and manual elimination of spurious fibers) by using a region of avoidance to only include the spinal cord. In addition, the whole post-processing time was limited to 15 min.

### Limitations and future directions

In the present study, the clinical application of tractography for spinal cord disease management is illustrated, providing additional information that standard anatomical MR sequences did not: supraselective site of fiber destruction, spinal cord deformation, tumor location within the spinal cord segment, etc. In cases reported here, we are able to demonstrate the interest of tractography for intramedullary cavernous malformation by identifying hematoma from spinal cord fibers, and therefore adapting the spinal cord approach. We also obtained a readable tractography in a patient with titanium for intervertebral fusion after spinal cord injury, thus reporting an efficient method of spinal cord tractography even if native DTI images contained many artifacts. Moreover, recent studies on spinal cord injury showed that tractography can be a helpful tool in the assessment of the severity of the damage, and to predict patient prognostic (Zhu et al., 2021; Costanzo et al., 2022). Finally, as proposed by other authors, tractography helped physicians in the diagnosis of intramedullary tumors, according to the fiber's location (Ducreux et al., 2006). However, only three cases were reported herein. These are in addition to the few cases reported (Filippi et al., 2010; Choudhri et al., 2014; Dauleac et al., 2019a), but the added value of tractography in terms of prognosis after injury but also the safety of intramedullary surgery should be assessed in a large prospective cohort of subjects, which we have initiated. In addition, this full tractography method should be applied further to the thoracic spinal cord and conus medullaris.

The software used herein—DSI Studio—was preferred for its simplicity and the combination of all tractography steps in one tool but there remains a learning curve. With advances in computer science, post-processing could be fully automated, including machine learning for tumor segmentation and region of avoidance design; stitching of acquisition boxes for cervical/thoracic/lumbar tractograms (Wang et al., 2012a); and multiband MR acquisition in reversed-phase encoding to double the spatial and angular resolution (Idiyatullin et al., 2015).

Full cervical cord tractography currently appears as a condensation of tracts without differentiation from one another. The output of the described pipeline demonstrates streamlines coursing through all of the gray matter in a cranio-caudal direction, indistinguishable from the adjacent white matter tracts. One of the next challenges is therefore the differentiation of spinal tracts within the spinal cord. In this way, the “Spinal Cord Toolbox” (De Leener et al., 2017) appears to be an interesting tool. This software package proposes a segmentation of the white and gray matter, and a probabilistic atlas of the spinal tracts (De Leener et al., 2017). This atlas could be superimposed on an axial view of a full spinal cord tractography to depict gray central “butterfly” matter and peripheral white fibers. However, this atlas was built at the cervical level, with extrapolation to the entire spinal cord. Since the exact location of the tracts might change across levels and subjects (especially in patients with intramedullary lesion), a “tailored” atlas of each spinal cord level should be built. Our team is currently working on this (NCT05079945).

This simplified spinal cord tractography protocol is aimed to enable its use in routine practice. As for brain tractography, which can be incorporated into a neuronavigation system, it could be helpful for neurosurgeons to see the spinal cord tractograms merged with anatomical images (T2-weighted imaging) into an image guidance platform. However, it would require translating images obtained with DSI Studio back into neuronavigation systems. Implementing spinal cord tractography in navigation systems could also promote neuroanatomy teaching and presurgical planning. In addition, incorporating 3D-tractography rendering into augmented reality and virtual reality devices could help young neurosurgeons to better understand the anatomy and anticipate the pitfalls of such intramedullary surgery.

## Conclusion

This study reports a simplified tractography pipeline that reconstructs a full cervical cord tractography, which could be used in routine clinical practice. The easy-to-use method developed herein is accessible to any neurosurgeon who wishes to perform spinal cord tractography for patients with spinal cord injury, tumor, or compression.

## Data availability statement

The raw data supporting the conclusions of this article will be made available by the authors, without undue reservation.

## Ethics statement

The studies involving human participants were reviewed and approved by Ethics Committee of Hospices civils de lyon. The patients/participants provided their written informed consent to participate in this study.

## Author contributions

Conception and design: CD and TJ. Acquisition of data: CD, CN, and FC. Analysis and interpretation of data and drafting the article: CD, TJ, and CF. Critically revising the article and reviewed submitted version of manuscript: CD, CF, IP-G, F-CY, JF-M, FC, and TJ. Administrative/technical/material support: CF, CN, and FC. Study supervision: FC and TJ. All authors contributed to the article and approved the submitted version.

## Conflict of interest

The authors declare that the research was conducted in the absence of any commercial or financial relationships that could be construed as a potential conflict of interest.

## Publisher's note

All claims expressed in this article are solely those of the authors and do not necessarily represent those of their affiliated organizations, or those of the publisher, the editors and the reviewers. Any product that may be evaluated in this article, or claim that may be made by its manufacturer, is not guaranteed or endorsed by the publisher.

## Supplementary material

SUPPLEMENTARY VIDEO 1 Spinal Cord Full Tractography.

## Abbreviations

CSF: cerebrospinal fluid
DTI: diffusion tensor imaging
MR: magnetic resonance
ROI: region of interest.
